# Molecular characterization of extended-spectrum beta-lactamases (ESBLs) produced by clinical isolates of *Acinetobacter baumannii* in Saudi Arabia

**DOI:** 10.1186/s12941-015-0098-9

**Published:** 2015-08-20

**Authors:** Essam J. Alyamani, Mohamed A. Khiyami, Rayan Y. Booq, Basel M. Alnafjan, Musaad A. Altammami, Fayez S. Bahwerth

**Affiliations:** Molecular Bacteriology, National Center for Biotechnology, King Abdulaziz City for Science and Technology, P.O. Box 6086, Riyadh, 11442 Saudi Arabia; Herra General Hospital, Makkah, Saudi Arabia

**Keywords:** *Acinetobacter baumannii*, Phenotyping, Genotyping, Saudi Arabia

## Abstract

**Background:**

*Acinetobacter baumannii* is a common opportunistic pathogen that causes major nosocomial infections in hospitals. In this study, we hypothesized a high prevalence of *A. baumanni* ESBL (extended-spectrum beta-lactamase) among all collected isolates.

**Methods:**

*A. baumannii* isolates (n = 107) from ICU (Intensive care unit) of local hospitals in Makkah were phenotypically and genotypically characterized. The identity and antibiotic susceptibility of *A. baumannii* strains were determined using the Vitek-2 system. The identified ESBL producers were further analyzed by PCR and sequencing followed by MLST typing. *bla*_*TEM*_*, bla*_*SHV*_, and the *bla*_*CTX*-*M*-_group genes 1, 2, 8, 9, and 25 were investigated. Furthermore, *bla*_*OXA51*-*like*_ and *bla*_*OXA23*-*like*_ genes were also examined in the carbapenem-resistant *A. baumannii* isolates.

**Results:**

Our data indicated a high prevalence of *A. baumannii* ESBL producers among the collected strains. Of the 107 *A. baumannii* isolates, 94 % were found to be resistant to cefepime and ceftazidime, and aztreonam using the Vitek 2 system. The genes detected encoded TEM, OXA-51-like and OXA-23-like enzymes, and CTX-M-group proteins 1, 2, 8, 9, and 25. MLST typing identified eight sequence type (ST) groups. The most dominant STs were ST195 and ST557 and all of them belong to worldwide clonal complex (CC) 2.

**Conclusions:**

This study has shown that there is a high prevalence of antimicrobial resistance in *A. baumannii*. The diversity of STs may suggest that new ESBL strains are constantly emerging. The molecular diversity of the ESBL genes in *A. baumannii* may have contributed to the increased antimicrobial resistance among all isolates.

## Background

*Acinetobacter baumannii* is an opportunistic and rapidly emerging pathogen. It is an important agent of nosocomial infections worldwide, such as urinary tract infections, septicemia, pneumonia, burns, meningitis, and wound infections in hospitals, due to its remarkable propensity to rapidly acquire resistance determinants to a wide range of antibacterial agents [1–4]. Many studies have documented high rates of multidrug-resistance (MDR) in *A. baumannii* [4–6]. The development of resistance to the third-generation cephalosporins was a major breakthrough in the fight against MDR strains. However, due to the frequent use of these agents, new plasmids encoding β-lactamase capable of hydrolyzing extended-spectrum cephalosporins were first reported in 1983 [7, 8]. These extended-spectrum β-lactamases (ESBLs) are mutant, plasmid-mediated, and produced by gram negative bacilli that mediate resistance to penicillin, cephalosporins, and monobactams [9]. These ESBLs are commonly recognized in Enterobacteriaceae, *Pseudomonas aeruginosa*, and *Acinetobacter baumannii* and are found worldwide [10]. The majority of ESBLs are members of either the TEM, SHV, or CTX-M (class A) families based on the Ambler molecular classification of β-lactamase genes [11, 12]. One of the major genes of ESBL family is the CTX-M, which is divided into five phylogenetic groups based on amino acid sequence identity: the CTX-M-1 group, the CTX-M-2 group, the CTX-M-8 group, the CTX-M-9 group, and the CTX-M-25 group. The presence and prevalence of these different groups are variable depending on the geographical locale [13, 14]. In Saudi Arabia, the high prevalence of ESBL *A. baumannii* was reported in several studies [15–17]. The PCR technology is widely used technique to screen for ESBL in modern hospitals. A specific multiplex PCR assay has been optimized to screen for multiple ESBL genes to facilitate and monitoring the spread and emergence of ESBL-producing bacteria [18]. The epidemiologic characterization of *A. baumannii* by multilocus sequence typing (MLST) is a highly used method and has been applied successfully [19]. With reports on the high prevalence of ESBL production in members of *A. baumannii* globally and a paucity of information specifically regarding the emergence of ESBL *A. baumannii* in major Saudi general hospitals in Makkah, this study reports the analysis of the antibiotic susceptibility profiles and molecular characterization of 107 *A. baumannii* ESBL producers isolated from ICU ward based on the phenotypic and genotypic approach. Understanding the molecular nature of the spread of *A. baumannii* in local hospitals is important, especially in hospitals that admit thousands of local and foreign people during their holy journey to Makkah. This work may enhance our understanding of the extent of the epidemiologic re-emergence of this bacterium. The genes that were investigated from *A. baumannii* isolates by PCR were *bla*_*TEM*_, *bla*_*SHV*_, and the *bla*_*CTX*-*M*-_group genes 1, 2, 8, 9, and 25. Furthermore, *bla*_*OXA51*-*like*_ and *bla*_*OXA23*-*like*_ enzymes were examined in carbapenem-resistant *A. baumannii.* This work may partially contribute to the global effort to map the molecular signature of *A. baumannii*.

## Methods

### Study design

A total of 107 bacterial isolates were collected from different ICU patients from clinical labs at local general hospitals in Makkah during 2 years from 2012 to 2014. Samples were subjected to a conventional microbiology analysis, phenotyping, and genotyping characterizations at the national center for biotechnology, KACST. The nature of the samples were blood, and skin wound infections predominantly.

### Species identification and antimicrobials susceptibilities

Bacterial identities were confirmed using the Vitek 2 system (GN ID Card, bioMérieux, Craponne, France) and PCR. Antibiotic susceptibility testing was conducted according to the manufacturer’s recommendations (gram negative antimicrobial susceptibility testing (AST) cards, bioMérieux, Craponne, France). The extraction of genomic DNA was performed using QIAGEN kits (QIAamp DNA Mini Kit, cat# 69506, QIAGen, Valencia, CA, USA) according to the manufacturer’s recommendations and or the MagNA Pure *LC* DNA Isolation Kit *III* Bacteria, Fungi (Roche, Basel, Switzerland). The results of Vitek ESBL susceptibility test were reported according to the CLSI criteria. Quality-control bacterial strains (*E. coli* ATCC 35218 and *Pseudomonas aeruginosa* ATCC 27853) were used in all tests.

### Detection of ESBL and carbapenem genes by PCR

All of the positive ESBL isolates according to phenotypic assays (n = 100) were further confirmed by PCR and sequencing. The genes investigated in this study were the *bla*_*TEM*_*, bla*_*SHV*_, and *bla*_*CTX*-*M*-_group genes 1, 2, 8, 9, and 25. Furthermore, *bla*_*OXA51*-*like*_ and *bla*_*OXA23*-*like*_ enzymes were tested for carbapenem-resistant *A. baumannii.* The gDNA was extracted using a QIAamp Genomic DNA kit (QIAGEN, Venlo, Netherlands) and used for PCR directly, or overnight cultures were boiled at 95 °C for 10 min to produce a bacterial gDNA/plasmid lysate that was diluted 1:10 with ddH_2_O before it was used for PCR. PCR amplification was performed with either 1 µl of pure gDNA or 10 µl of gDNA/plasmid lysate as a template. Final reactions of 25 μl of illustra PuReTaq Ready-To-Go PCR beads (GE Health Biosciences, USA) were used in the PCR reaction according to the manufacturer’s recommendations. The reactions were set up as follows: 10–22 μl of nuclease-free water (Promega) depending on the DNA templates being used; 2 µl of 10 pmol of each *bla*_*TEM*_*, bla*_*SHV*_, and *bla*_*CTX*-*M*-_group genes 1, 2, 8, 9, and 25; *bla*_*OXA51*-*like*_ and *bla*_*OXA23*-*like*_ forward and reverse primers (Eurofins MWG Operon, Germany); and 1–10 µl of DNA template or bacterial lysate were used (Table 1) [18]. The cycling conditions of the PCR are illustrated in Table 1. All of the amplicons were size fractionated using 1 % agarose gel electrophoresis and visualized under ultraviolet illumination using the Gel Doc EZ system (Bio-Rad, Hercules, CA, USA).

**Table 1:** Primers for the rapid characterization of *A. baumannii* by multiplex PCR

No.	blaOXA-like enzymes of *A. baumannii*			Amplification conditions
1	blaOXA-51 F	5′-TAA TGC TTT GAT CGG CCT TG	353 bp	Initial denaturation at 94 °C for 5 min, followed by 30 cycles of 94 °C for 25 s, 52 °C for 40 s and 72 °C for 50 s, and a final elongation at 72 °C for 6 min
2	blaOXA-51R	5′-TGG ATT GCA CTT CAT CTT GG		
3	blaOXA-23-F	5′-GAT CGG ATT GGA GAA CCA GA	501 bp	
4	blaOXA-23-R	5′-ATT TCT GAC CGC ATT TCC AT		
blaCTX-M genes				
7	Group 1-F	5′-AAA AAT CAC TGC GCC AGT TC	415 bp	Initial denaturation at 94 °C for 5 min, followed by 30 cycles of 94 °C for 25 s, 52 °C for 40 s and 72 °C for 50 s, and a final elongation at 72 °C for 6 min
8	Group 1-R	5′-AGC TTA TTC ATC GCC ACG TT		
9	Group 2-F	5′- CGA CGC TAC CCC TGC TAT T	552 bp	
10	Group 2-R	5′-CCA GCG TCA GAT TTT TCA GG		
11	Group 9-F	5′-CAA AGA GAG TGC AAC GGATG	205 bp	
12	Group 9-R	5′-ATT GGA AAG CGT TCA TCA CC		
13	Group 8F	5′-TCG CGT TAA GCG GAT GAT GC	666 bp	
14	Group 8R	5′-AAC CCA CGA TGT GGG TAG C		
15	Group 25F	5′-GCA CGA TGA CAT TCG GG	327 bp	
16	Group 25R	5′-AAC CCA CGA TGT GGG TAG C		
1	TEM-F	5′-CATTTCCGTGTCGCCCTTATTC	800 bp	Initial denaturation at 94 °C for 10 min, followed by 30 cycles at 94 °C for 40 s, 60 °C for 40 s, and 72 °C for 1 min, and a final elongation step at 72 °C for 7 min
2	TEM-R	5′-CGTTCATCCATAGTTGCCTGAC		
3	SHV-F	5′-AGCCGCTTGAGCAAATTAAAC	713 bp	
4	SHV-R	5′-ATCCCGCAGATAAATCACCAC		
1	16S rRNA 8F	5′-GCG GAT CCG CGG CCG CTG CAG AGT TTG ATC CTG GCT CAG	797 bp	Initial denaturation at 94 °C for 5 min, followed by 35 cycles at 94 °C for 60 s, 55 °C for 30 s, and 72 °C for 60 s, and a final elongation step at 72 °C for 7 min
2	16S rRNA 805R	5′-GCG GAT CCG CGG CCG CGG ACT ACC AGG GTA TCT AAT		

### Amplification and sequencing of the 16S rRNA gene

Amplification and sequencing of 16S rRNA were performed to confirm the identity of *A. baumannii* used in this study [20]. In the PCR amplification, each reaction contained 25 μl of illustra PuReTaq Ready-To-Go PCR beads (GE Health Biosciences, USA). The reaction was set up as follows: 22 μl of nuclease-free water (Promega), 2 µl of 10 pmol of each forward and reverse primer (Eurofins MWG Operon, Germany) were used (Table 1) [18]. Exactly 1 μl of 100 ng/µl DNA template was added to the beads. The amplification conditions are highlighted in Table 1. The amplification products were subjected to gel electrophoresis in 1 % agarose followed by ethidium bromide staining and were visualized under ultraviolet illumination using the Gel Doc EZ system (Bio-Rad, Hercules, CA, USA). Sense and anti-sense strands of PCR amplicons were purified and sequenced in an ABI 3130 Genetic Analyzer (Life Technologies, Carlsbad, CA, USA) using ABI BigDye terminator cycle sequencing ready reaction kit chemistry according to the manufacturer’s recommendations. Following sequencing, the data were identified using a basic local alignment search tool BLAST-n (http://www.ncbi.nlm.nih.gov/BLAST) or RDP database [21]. The identification of *A. baumannii* using the 16S rRNA was unequivocal. Therefore, there was no need to use additional confirmatory targets such as *rpoB* and *gyrB* genes [22].

### Multilocus sequence typing (MLST)

The *Acinetobacter baumannii* complex MLST typing was performed by utilizing seven house-keeping genes: Citrate synthase (*gltA*), DNA gyrase subunit B (*gyrB*), Glucose dehydrogenase B (*gdhB*), Homologous recombination factor (*recA*), 60-kDa chaperonin (*cpn60*), Glucose-6-phosphate isomerase (*gpi*), RNA polymerase sigma factor (*rpoD*). The primers used for amplification and sequencing are illustrated in Table 2 [19]. The PCR amplifications were completed in a MasterCycler nexus (Eppendorf, Hamburg, Germany) with the following conditions: 35 cycles of initial denaturation at 94 °C for 5 min, followed by 35 cycles of denaturation at 94 °C for 1 min, annealing at 55 °C for 1 min, and extension at 72 °C for 2 min and 4 min final extension at 72 °C. The PCR products were directly verified by 1 % agarose gel electrophoresis before they were purified from the reaction mixture for sequencing. Bidirectional sequencing was performed for each isolate. Different allele sequences were assigned for each locus with an arbitrary allele number for identification. Each bacterial isolate was characterized by a pattern of numbers defining its sequence type (ST). The sequences of the seven housekeeping genes were analyzed by using an *A. baumannii* database (http://pubmlst.org/abaumannii/) [23]. The allelic profile similarities were produced by BioNumerics version (7) created by Applied Maths NV. Available from (http://www.applied-maths.com).

**Table 2:** Primers used in PCR to amplify the seven housekeeping genes in *A. baumannii* isolates

No.	Locus	Primer	Sequences	Amplicon size (bp)	Usage
1	*gltA*	Citrato F1	AAT TTA CAG TGG CAC ATT AGG TCC C	722	Amp/seq
		Citrato R12	GCA GAG ATA CCA GCA GAG ATA CAC G		Amp/seq
2	*gyrB*	gyrB_F	TGA AGG CGG CTT ATC TGA GT	594	Amp/seq
		gyrB_R	GCT GGG TCT TTT TCC TGA CA		Amp/seq
3	*gdhB*	GDHB 1F	GCT ACT TTT ATG CAA CAG AGC C	774	Amp
		GDH SEC F	ACC ACA TGC TTT GTT ATG		Seq
		GDHB 775R	GTT GAG TTG GCG TAT GTT GTG C		Amp
		GDH SEC R	GTT GGC GTA TGT TGT GC		Seq
4	*recA*	RA1	CCT GAA TCT TCY GGT AAA AC	425	Amp/seq
		RA2	GTT TCT GGG CTG CCA AAC ATT AC		Amp/seq
5	*cpn60*	cpn60_F	GGT GCT CAA CTT GTT CGT GA	640	Amp/seq
		cpn60_R	CAC CGA AAC CAG GAG CTT TA		Amp/seq
6	*gpi*	gpi_F	GAA ATT TCC GGA GCT CAC AA	456	Amp/seq
		gpi_R	TCA GGA GCA ATA CCC CAC TC		Amp/seq
7	*rpoD*	rpoD-F	ACC CGT GAA GGT GAA ATC AG	672	Amp/seq
		rpoD-R	TTC AGC TGG AGC TTT AGC AAT		Amp/seq

### Ethics statement

Ethical approval and consent were not required for this project because no human nor animal subjects were used.

## Results

### Antimicrobial susceptibility testing and screening for ESBL

**Fig. 1:**
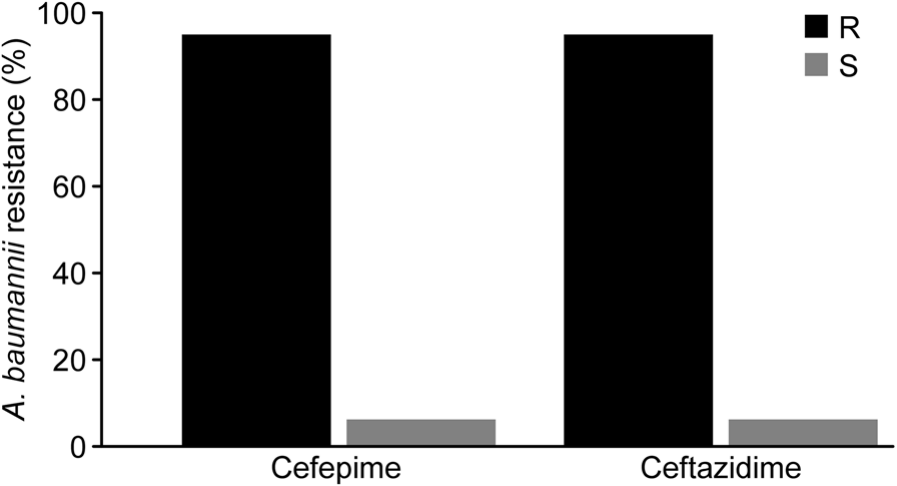
Cephalosporin susceptibility pattern by *A. baumannii* isolates. Among the 107 isolates of *A. baumannii* tested, 100 isolates (94 %) were confirmed as ESBL producers by phenotypic assay

**Fig. 2:**
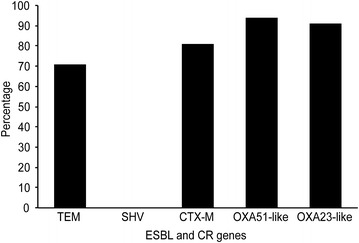
The overall distribution of ESBL and carbapenemase genes detected in *A. baumannii* isolates

In this study, 94 % (100/107) of *A. baumannii* were MDR. Among the 107 isolates of *A. baumannii* tested, one hundred isolates were confirmed as ESBL producers by phenotypic and genotypic assays, four isolates were susceptible to the third generation cephalosporins (Figs. 1, 2) and three isolates were not confirmed as *A. baumannii* by 16S rRNA PCR. The ESBL *A. baumannii* were recovered from different clinical specimens, blood, and skin wound infections predominantly. The susceptibility data of the ESBL-producing *A. baumannii* showed that 94 % of the 107 isolates resistant to the panel of the VITEK 2 gram negative Susceptibility Card, whereas 4 % were sensitive isolates based on CLSI criteria.

## 16S rRNA identification and the detection of ESBL and carbapenemase genes

The 16S rRNA sequencing of all isolates (n = 107) generated a high score (≥97 % in total) of *A. baumannii* identity using the BLAST and Ribosomal Database Project (http://rdp.cme.msu.edu/) [21, 24]. To determine the extent of genotypic diversity among the MDR *A. baumannii*, PCR and sequencing of *bla*_*TEM*_, *bla*_*SHV*_, and the *bla*_*CTX*-*M*-_group genes 1, 2, 8, 9, and 25 and the *bla*_*OXA51*-*like*_, and *bla*_*OXA23*-*like*_ genes were employed. All of the PCR-based ESBL-positive *A. baumannii* isolates (n = 100) were concordant with the phenotyping data. Of these isolates, seventy-one (71 %) harbored the *bla*_TEM_ gene. None of them contained the *bla*_SHV_ gene and eighty-one isolates (81 %) encoded *bla*_CTX-M_-group genes 1, 2, 8, 9, and 25. Finally, ninety-four (94 %) isolates carried the carbapenemase gene OXA51-like, and ninety-one isolates (91 %) contained OXA23-like (Table 3; Fig. 2). The sequencing analysis of all of the genes showed approximately 90 % sequence similarity to the submitted sequences that are related to the genes deposited in GenBank.

**Table 3:** Detection and ESBL genotyping of 107 *Acinetobacter baumannii* clinical isolates

PCR size	501 bp	353 bp	713 bp	800 bp	327 bp	205 bp	666 bp	552 bp	415 bp
Gene	CTX-M1	CTX-M2	CTX-M8	CTX-M9	CTX-M25	TEM	SHV	OXA- 51-like	OXA-23-like
Positive isolates of 107 isolates	9	73	72	10	61	73	0	100	97
Percentage (%)	81					71	0	94	91

### Multilocus sequence typing analysis

**Fig. 3:**
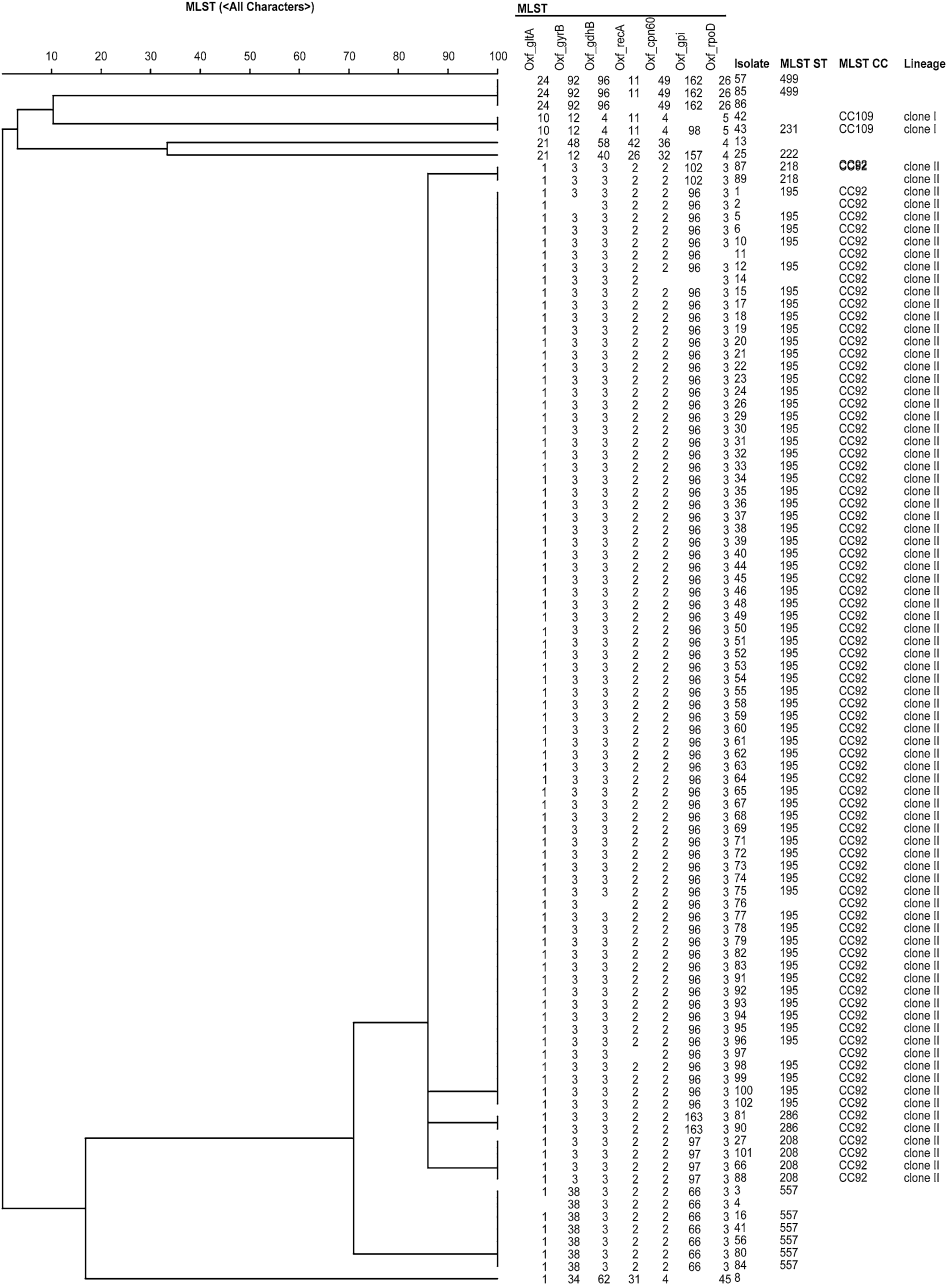
UPGMA (unweighted pair group method with arithmetic mean) dendrogram based on the catagorical coefficient applied to the allele IDs. All isolates with at least six loci amplified were included. The dendrogram was generated by BioNumerics 7 software. The ST numbers assigned for each isolate were generated by the Pasteur MLST scheme (http://pubmlst.org/abaumannii/). The tree is a rooted based on the nucleotide sequence of the six and seven housekeeping genes. The analysis was based on data sets that include all STs in the Pasteur MLST databases. The first clade consists of ST195, 208, 218 and 286; the second clade of ST231; the third clade of ST499; the fourth clade of ST 557; the fifth clade of ST222. The sixth and seventh clades have two nontypeable isolates due to low quality sequencing trace files

**Fig. 4:**
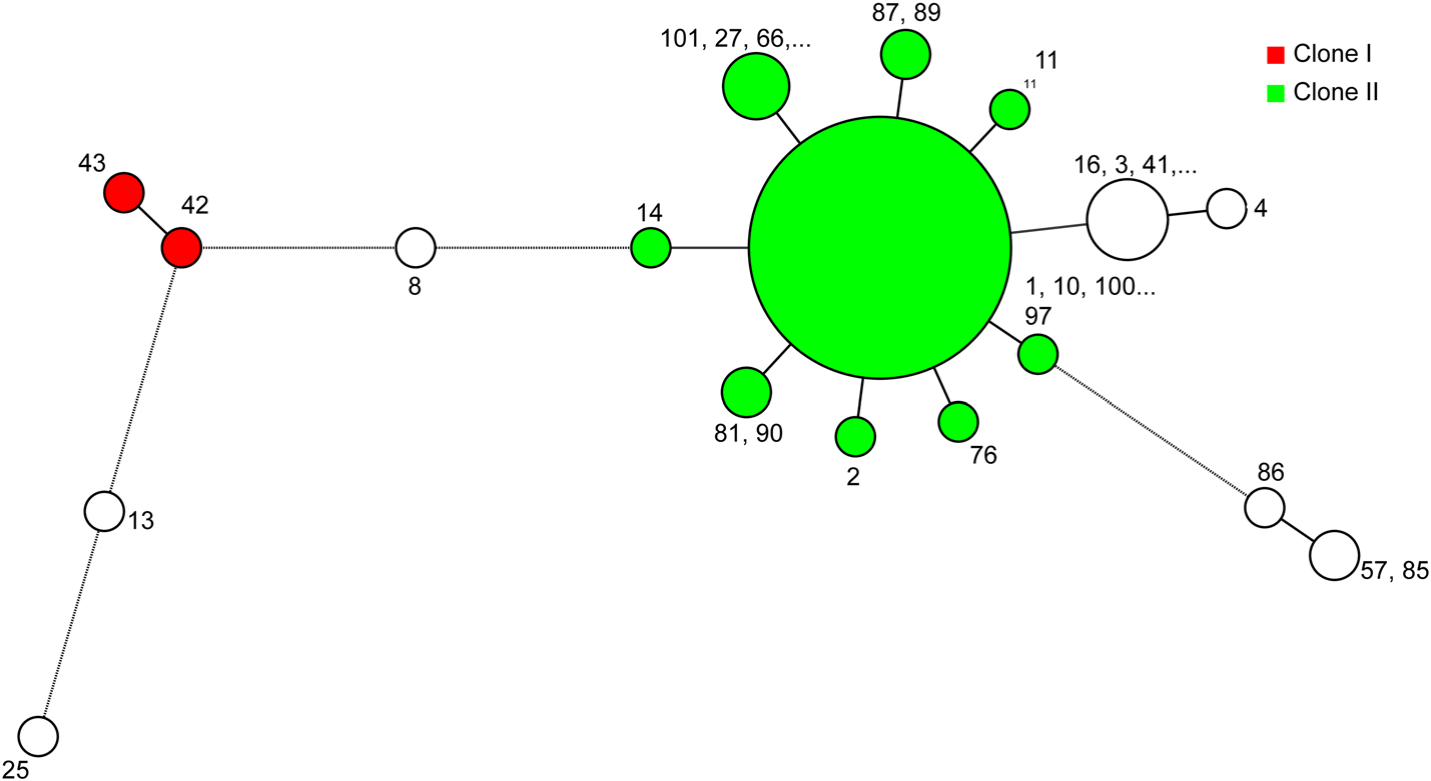
Minimum spanning tree constructed based on the allele IDs

MLST and sequence-based typing of ESBL and carbapenemase isolates were performed to analyze the genetic relationship of all of the isolates. The MLST anaylsis contains 97 isolates of 102. Five isolates were not typable due to low quality traces files and were not assigned STs but were included in the dendrogram. The MLST analysis allowed us to group the *A. baumannii* isolates into eight STs (Figs. 3, 4). MLST typing showed that the most dominant sequence type was ST195 (n = 69), followed by ST557 (n = 6), ST 208 (n = 4), ST499 (n = 2), ST218 (n = 2), ST231 (n = 1), ST222 (n = 1), and ST286 (n = 2). All of STs except ST 231 belonge to clonal complexity 2 (CC2) and lineage clone 2. The tree (Fig. 3) is based on the nucleotide sequence of at least 6 or 7 housekeeping genes. The analysis was based on data sets that include all STs in the Pasteur MLST databases of *A. baumannii* (http://pubmlst.org/abaumannii/).

## Discussion

In this study, we detected and characterized the phenotypic and genotypic nature of ESBL producers in *A. baumannii*, which were isolated from general hospitals in Makkah, Saudi Arabia. At least 107 *A. baumannii* isolates were characterized by the Vitek-2 system and PCR-sequencing followed by MLST typing. Our data indicated a high prevalence of *A. baumannii* ESBL producers among the collected isolates. A remarkable outcome of this study was the large number of antibiotic resistance genes found in these isolates. Ninety-four percent of *A. baumannii* isolates were found to have three major resistant determinants. We speculate that if more drug-resistant genes were screened, we would have found pan-resistant *A. baumannii* isolates.

CTX-M β-lactamases produced by *A.baumannii* strains is plasmid-mediated hence the wide spread and long time survival in hospitals. The CTX-M gene activity conferring resistance to cefotaxime and ceftazidime. We detected CTX-M group 1, 2, 8, 9, 25 in our current study (81 %). The high rate of prevalence of CTX-M resistance in gram negative bacteria may be influenced by mobile genetic elements around these genes which include transposon, insertion sequences (IS) and integrons [25]. Consistent with our study, all gram negative CTX-M producing bacteria are often associated with other families of other β-lactamases resistance causing multi-drug resistance phenomena. The high prevalence rate around the world of CTX-M makes it a predominant drug resistant gene in gram negative bacteria [26].

We studied the dynamic spread of *A.baumannii* in our population by MLST. The discriminatory power of the MLST system is comparable to other techniques such as pulsed field gel electrophoresis (PFGE). Yet, MLST provides a quick and easy method to study the epidemiology of ESBL-producing bacteria and to monitor the international emergence of multidrug resistant bacteria. Consistent with other studies that used MLST in the epidemiologic characterization of clinically important bacterial pathogens such as *A.baumannii*, *Streptococcus pneumoniae*, *Streptococcus pyogenes*, *Neisseria meningitidis*, *Campylobacter jejuni*, *Staphylococcus aureus*, *Enterococcus faecium*, *Haemophilus influenza*, and *Vibriocholera*, our study has detected different allelic diversity (STs) which belongs to clonal complex (CC)2 which is globally distributed in Europe, Asia, Africa, Australia, USA, South America [19, 27–29].

The drug of choice to treat nosocomial infection caused by *A.baumannii* is the carbapenems. However, there is an increasing rate of carbapenem-resistant *A.baumannii* around the world caused by OXA23-like enzume or OXA51-like enzyme acitivies [30]. The first OXA23-like enzyme with carbapenem-activity to *A. baumannii* was isolated and characterized in Scotland in 1985. This drug-resistant determinant is encoded by the plasmid therefore it is transferable [31]. This may explain the high prevalence of carbapenemase-producing *A. baumannii* in hospitals around the world. The other gene cluster in the OXA family is the *bla*_OXA-51_-like gene which is chromosomally encoded and naturally occurs in *A. baumannii*. The functional product of this gene delivers carbapenemase resistance to meropenem and imipenem; its role in carbapenem resistance may be influenced by the presence of IS*Aba1*. PCR mapping studies have found that the absence of this sequence upstream of blaOXA-51-like gene may contribute to a minimal effect on carbapenem susceptibility [32–36].

A recent study in the Gulf Countries Council (GCC) [37], namely, Saudi Arabia, the United Arab Emirates, Oman, Qatar, Bahrain, and Kuwait, suggested a high prevalence of carbapenemase resistance in *A. baumannii*, *Escherichia coli* and *Klebsiella pneumonia.A. baumannii* (n = 117) was studied as clusters in seven different sequence types: ST195, ST208, ST229, ST436, ST450, ST452 and ST499. Three of these sequences were identified in our study, including ST195, ST499, and ST208, which may suggest the circulation of these three STs in GCC countries [17, 37]. The circulation of the STs within GCC may be due to the closeness of these countries to each other. Recent reports have been accumulating from Saudi Arabia due to the wide and rapid spread of carbapenem-resistant gram negative bacteria isolated from local hospitals specially during high season [38–40].

The high level of detection of ESBL and carbapenemase resistance among local isolates may suggest an increasing incidence rate of infection with ESBL-producing *A. baumannii*. Such high rates of ESBL-producing bacteria may impose a burden on routine clinical practice, especially for patients with chronic diseases and immunocompromised patients. Although national surveillance data are lacking, outbreaks of infection due to ESBL-producing *A. baumannii* have been reported by many hospitals within the Kingdom of Saudi Arabia. The true prevalence of ESBL producers is not known and is likely underestimated because of the difficulties encountered in their detection by most local hospitals. However, it is clear that ESBL-producing bacteria are distributed worldwide and their prevalence is increasing [2, 6, 41, 42]. Therefore, periodic screening of ESBL-producing *A. baumannii* during the high hospital visitation season is recommended in all local hospitals to establish national surveillance data archives of the level of spread of ESBL producers.

## Conclusions

In this study, we randomly surveyed and characterized ESBL-producing *A. baumannii* from ICU of local hospitals in Makkah city, Saudi Arabia. Our data indicated a high prevalence of *A. baumannii* ESBL producers among the collected isolates. Based on MLST typing, we have evidence of eight STs groups in our isolates. The epidemiologic diversity of these isolates may suggest that new ESBL strains are constantly emerging. The molecular diversity of the ESBL genes in *A. baumannii* may have contributed to the increased antimicrobial resistance among all isolates. Therefore, periodic screening of ESBL-producing *A. baumannii* during the high hospital visitation season is recommended in all local hospitals.
